# Complications Following Spinal Caries, with Report of Cases

**Published:** 1895-07

**Authors:** Samuel Milliken

**Affiliations:** Surgeon-in-chief of the New York Infirmary for Crippled Children; Surgeon to the Infant, Children and Idiot’s Hospital, New York; 72 West Forty-ninth Street


### COMPLICATIONS FOLLOWING SPINAL CARIES, WITH REPORT OF CASES.

By SAMUEL MILLIKEN, M. D.,

Surgeon-in-chief of the New York Infirmary for Crippled Children; Surgeon to the Infant, Children and Idiot's Hospital, New York.

It is with the hope of preventing the dangerous and often fatal complications of Pott's disease, or tubercular spondylitis, that so much care is given to the mechanical treatment of these cases.

Early diagnosis, *i. e.*, before deformity exists, together with prompt and continued immobilization of the spinal column until ankylosis occurs and the inflammatory area has become quiescent or resolved, is the ideal result. Unfortunately the specialist does not see these cases very frequently in the incipiency of the disease, but on the contrary, a well-marked kyphosis is often present and not infrequently abscess and paralysis have to be dealt with. Of the following cases the first came to me from Bridgeport, Conn., February, 1889:

CASE I.—Raymond C., four years of age. Previous history obtained was that when two years and nine months old, the mother noticed that the child walked peculiarly and complained of pains in the stomach. This was in November, 1887. During February, 1888, the child became paralyzed in both lower extremities and one month later a spinal support was applied by the family physician. There was no improvement in the paralysis, although the brace was said to have been worn six months, when it was left off. Five months later, or one year after the paralysis developed, I saw the case. Examination.—There was a marked dorsal kyphosis with complete paralysis of the lower extremities and the usual atrophy that would be expected in such a case. The spine was immobilized and the patient kept recumbent. The saturated solution of iodide of potassium was ordered, beginning with 5 minims t. i. d. after meals and increased to 35 minims t. i. d. in the course of two weeks. The child was brought to me once a week so as to have the apparatus changed, and a bath given, thus preventing excoriations at the points of pressure. Within two weeks the toes could be moved voluntarily, and in May, 1889, three months after the treatment was begun, the child could walk. No further complications developed and in September, 1894, all sup port was left off. While the lower extremities are smaller than usual for a child nearly ten years old, he walks well and has perfect use of the lower extremities. The bodies of the vertebræ are firmly ankylosed, and seven months after all mechanical support was left off, there was no trouble.

CASE II.—Willie F., age three years, was brought to me March 31, 1891, by his uncle, a physician. A small kyphos had been noticed two months before. The child had previously suffered from rachitis. Examination.—He walked rather unsteadily, and the reflexes of the lower extremities were unduly excited. There was a distinct kyphosis in the upper dorsal region. Beginning paraplegia was diagnosed and within two weeks the lower limbs were almost entirely paralyzed. Under the most careful mechanical treatment, the apparatus being only changed by me, and the iodide of potassium continued, the patient walked in a little over a year. The inability of the patient to endure the increasing and continued large doses of iodide, owing to a weak digestive tract, accounts for the delay in recovering from the paralysis.

CASE III.—Edwin F. S., nineteen months of age, came under my observation June 13, 1891. When ten months old the child could stand with the assistance of a chair, but during the following winter he had chicken pox and subsequently bronchitis, and soon no inclination to stand was manifested. There was no tubercular history in the family. In addition to a well-marked mid-dorsal kyphosis, there was a psoas abscess on the right side, which produced a rectangular contraction of the thigh on that side. The spine was immobilized and operation on the abscess was deferred until September 2d, owing to the heat of the summer, when it was aspirated under chloroform anæsthesia and a little over two ounces of pus was evacuated. The abscess refilled within a few weeks, but the general health continued to improve and the child was able to walk with the assistance of a chair. On January 10, 1892, the patient died of diphtheria.

CASE IV.—Agnes W., five years of age, referred to me by Dr. Ferdinand King, of New York, May 25, 1893, illustrates the difficulties in diagnosing the disease when situated in the lumbo-sacral region. At two of the large hospitals of this city, a diagnosis of sprain was made, as a history of traumatism was given. Although the kyphos was small, there was a distinct psoas abscess on the left side.

CASE V.—Lester M., three years of age, came to me August 9, 1893, giving a history of trouble with the spine for more than a year. The knuckle in the upper dorsal region was easily made out and a large abscess was found on the right side of the neck. The patient had worn braces and jackets during the year, but was not under the care of any one surgeon for more than a few months. October 3, 1893, the abscess was evacuated under ether anæsthesia, the spine in the meantime having been kept immobilized. My last note of the case was March 21, 1895, when a second spinal brace was applied. The general health was good and the abscess was closed.

Conclusions.—In drawing conclusions from these notes taken at random from my cases, it is readily seen that the complications of Pott's disease are largely due to a delay in making the diagnosis and faulty mechanical treatment. While paralysis is possibly the most alarming to the family, a more favorable prognosis can be offered than from abscess. Perfect immobilization of the spine is essential in preventing deformity and other complications. The paraplegia from compression myelitis is rarely due to displacement of the bone, but to the inflammatory deposit. Post-mortem and clinical facts are abundant to prove this assertion, and the increasing doses of the iodide of potassium is the best means of producing absorption of the pathogenic material. Deposits in the other parts of the body are liable to take place, but tubercular meningitis is less frequent in occurrence in case of spinal caries, than is met with in joint disease of the lower extremities produced by the bacillus of tuberculosis.

*72 West Forty-ninth Street.*

